# Functional Magnetic Resonance Imaging of Brain Function and Emergence Agitation of Patients with Dexmedetomidine-Assisted General Anesthesia under Comfortable Nursing Intervention

**DOI:** 10.1155/2022/8527568

**Published:** 2022-07-19

**Authors:** Jingnan Gao, Qiao Zheng, Mingmin Liu, Jie Bao

**Affiliations:** Department of Anesthesiology, First Affiliated Hospital with Nanjing Medical University, Nanjing 201129, Jiangsu, China

## Abstract

In order to explore the effects of dexmedetomidine (DEX) on functional magnetic resonance imaging (fMRI) and emergence agitation of patients who underwent general anesthesia surgery with sevoflurane under comfortable nursing intervention, 66 patients who received upper abdominal surgery were selected as research objects. According to nursing and anesthesia methods, the patients were randomly divided into control group (routine nursing and anesthesia), group A (routine nursing and DEX-assisted anesthesia), and group B (comfortable nursing and DEX-assisted anesthesia). The differences in the brain fMRI characteristics, hemodynamic indexes, anesthesia recovery indexes, and nursing satisfaction in the perioperative period were evaluated. The results showed that the regional homogeneity values were different in different brain regions, but there was no difference in the *Z* value of functional connectivity(*P* > 0.05). Compared with the control group, heart rate, mean arterial pressure, awakening time, extubation time, the Riker sedation-agitation scale (SAS) score, and anesthetic dosage were signally decreased in group A and group B, while the Ramsay scores, the postanesthesia care unit (PACU) stay, and anesthesia maintenance time in the two groups was obviously increased(*P* < 0.05). Compared with group A, the extubation time, the SAS score, PACU stay, and hospital stay were all remarkably reduced in group B, while the nursing satisfaction score was greatly increased(*P* < 0.05). To sum up, DEX was helpful to safely and effectively reduce the occurrence of emergence agitation in patients under general anesthesia surgery with sevoflurane. Besides, comfortable nursing intervention could further reduce the incidence of emergence agitation in patients with general anesthesia, shorten the length of hospital stay, and improve nursing satisfaction.

## 1. Introduction

Sevoflurane has the characteristics of rapid induction, rapid recovery, and stable efficacy, which has been widely used in clinical anesthesia [1]. Due to surgical trauma, general anesthesia, endotracheal intubation stimulation, and environmental factors, patients under sevoflurane anesthesia have a high probability of complications such as emergence agitation [2, 3]. To reduce the incidence of complications such as the emergence agitation, chills, and hypotension in patients with general anesthesia, it is necessary to use a certain dose of sedative and analgesic drugs to relieve clinical symptoms [4]. Dexmedetomidine (DEX) is a new type of highly selective *α*2 adrenergic receptor agonist, which can inhibit the excitability of the sympathetic nervous system and reduce the level of catecholamines in the blood [5]. DEX anesthesia can relieve respiratory depression [6]. Hence, the effect of DEX on the prevention of emergence agitation in patients has become the focus that is concerned by anesthesiologists. To ensure the safety of patients undergoing surgery, it is very important to strengthen the nursing management of patients to ensure the life safety of patients and improve the overall nursing effect and treatment effect. Comfortable nursing is one of the most used nursing models in clinical practice. This nursing model is patient-centered and meets the psychological and physiological needs of patients to the greatest extent. Through the implementation of respiratory nursing, complication prevention, psychological nursing measures, etc., it can effectively adjust the adverse emotions of patients and improve the postoperative recovery effect.

Functional magnetic resonance imaging (fMRI) is a new imaging method for exploring the brain function of patients, which has the characteristics of noninvasive and high time-spatial resolution [7]. Blood oxygen level-dependent fMRI (BOLD-fMRI) is widely applied in clinical practice. A patient's brain neurons become excited, which can cause an increase in cerebral blood flow (oxygen and hemoglobin). When the brain oxygen consumption level is lower than the blood flow level, the deoxygenated hemoglobin levels are reduced [8, 9]. Deoxygenated hemoglobin is a paramagnetic substance, and a decrease in its content can cause an enhancement of T2-weighted imaging (T2WI) signals. Accordingly, T2WI can reflect the activity state of local brain neurons in patients [10]. Functional connectivity is an index to measure the correlation of time series in BLOD-fMRI signals, which can reflect the synchronization of the functional activities of patients' brain regions [11].

However, there are relatively few investigations on the effects of anesthesia methods on patients' brain function. This study was to explore the preventive effect of comfortable nursing combined with DEX on postoperative brain BLOD-fMRI characteristics and the emergence of agitation in patients undergoing general anesthesia by sevoflurane. Patients who underwent upper abdominal surgery were selected as research objects. The effects of routine nursing and comfortable nursing combined with DEX-assisted anesthesia on brain functional connectivity and emergence agitation were explored. It aimed to provide a reference for the rational use of general anesthesia in patients during clinical surgery.

## 2. Materials and Methods

### 2.1. Research Objects

In this study, 66 patients who underwent elective upper abdominal surgery in the hospital from May 2019 to May 2021 were selected as the research objects. There were 32 male patients and 34 female patients. The patients were randomly divided into control group, group A, and group B, with 22 patients in each group. Patients in the control group were not given DEX anesthesia under routine nursing intervention. Patients in group A received DEX anesthesia induction under routine nursing intervention. Patients in group B were given DEX anesthesia induction under comfort nursing intervention. The informed consent of all patients was obtained, and the procedure of this study had been approved by the ethics committee of the hospital.

The inclusion criteria were as follows. (1) Patients whose ages ranged from 20 to 65 years old. (2) Patients who were classified as degree I or II by the *American Society of Anesthesiology Engineers* (ASA). (3) Patients with body mass index in the range of 18 to 30 kg/m^2^. (4) Patients without serious cardiovascular and cerebrovascular diseases. (5) Patients with normal internal organ function. The exclusion criteria were as follows. (1) Patients who were classified as degree III or IV by ASA. (2) Patients with diabetes, hypertension, hyperthyroidism, or neuropsychiatric diseases. (3) Patients with heart failure, liver or kidney failure, sepsis, and other diseases that seriously affected body function. (4) Patients with a history of allergy to *α*2 adrenergic receptor agonist; (5) Patients who were unable to articulate their thoughts.

### 2.2. Anesthesia Methods

Before surgery, fasting and water deprivation were required, routine electrocardiogram (ECG) monitoring was performed, and peripheral venous access was established. 15 min before surgical anesthesia, patients in group A and group B were induced by continuous intravenous pumping of DEX at 1 *μ*g/kg/h. Patients in the control group were intravenously pumped with 0.9% NaCl solution at the same speed. After patients entered the operating room, they received oxygen through a mask with an oxygen flow rate of 6 L/min. The anesthetic concentration of sevoflurane was adjusted to 8% for anesthesia induction. Subsequently, 1 *μ*g/kg remifentanil was injected slowly into the peripheral vein for 1 min. The concentration of sevoflurane was adjusted to 1% for ventilation assistance based on CO_2_ partial pressure. During the surgery, sevoflurane concentration needed to be adjusted according to the indexes, and remifentanil needed to be injected intermittently.

### 2.3. Nursing Intervention

For routine nursing intervention, patients were examined for relevant indexes, and health education was given to patients before the surgery. Patients were informed of surgical anesthesia methods, treatment methods, precautions, and possible postoperative complications.

For comfortable nursing intervention, the changes in vital signs and consciousness state of patients in each group were closely observed, and the corresponding treatment was performed according to the classification of agitation. The psychological nursing of patients needed to be strengthened before surgery. Nursing staff needed to be kind, patient, and detailed to introduce the condition, surgical methods, and use of equipment to patients. The operating room temperature was controlled at about 25°C. After the operation was over and patients were transferred to the ward, it was necessary to help patients turn over in time with gentle movement. Besides, according to the actual situation of patients, the tightness of the restraint band was adjusted to prevent the occurrence of postoperative venous thrombosis and other complications. In the process of postoperative recovery, nursing staff needed to pay attention to patients' rehabilitation state at all times and patiently comfort patients' bad emotions to relieve their tension, anxiety, and fear. Simultaneously, nursing staff needed to help patients with the best state to spend the agitation period after general anesthesia surgery. After extubation, sputum suction was performed in time to reduce the incidence of complications caused by choking when patients fully breathed autonomously.

### 2.4. Collection of Resting-State MRI Data

3.0T magnetic resonance equipment was adopted for the collection of brain images. The patient was placed in a supine position on the scanning bed, and the head was fixed with a spongy cushion. The image scanning was performed in a relaxed and awake state of patients. The resting-state kinetic MRI data were obtained by gradient-echo sequence. The parameters were set as follows. The repetition time was 2,000 ms, the echo time was 30 ms, the scanning field of vision was 240*∗*240 mm, the matrix size was 64*∗*64, the in-plane resolution was 3.75*∗*3.75 mm^2^, the layer thickness was 3.0 mm, the number of layers was 40, the flip angle was 90°, and the scanning duration was 8 min. The imaging parameters of the T1-weighted structure diagram of the patient were collected. The repetition time was 9 ms, the echo time was 3.2 ms, the scanning field of vision was 256*∗*256 mm^2^, the matrix size was 512*∗*512, the in-plane resolution was 0.5*∗*0.5 mm^2^, the layer thickness was 1 mm, the number of layers was 192, and the flip angle was 9°.

The image data in the digital imaging and communication in medicine (DICOM) format was converted to the neuroimaging informatics technology initiative (NIFTI) format, and the time layer and head range were corrected. The fMRI images were standardized to narrow differences in shape and size of the brain among individuals. Then, the space smoothing in the image was implemented by using an 8*∗*8*∗*8 mm^3^ half-height full width. Finally, the image was processed by low pass filtering in the 0.01–0.08 Hz frequency band.

### 2.5. Observation Indexes

For the usage amount of anesthetic drugs, the dosage of remifentanil and sevoflurane in each group was recorded during treatment.

For vital signs, changes in heart rate (HR) and mean arterial pressure (MAP) before anesthesia (T0), before DEX anesthesia (T1), after administration of DEX (T2), at the time of extubation (T3), 5 min after extubation (T4), 15 min after extubation (T5), and 30 min after extubation (T6) in each group were all recorded.

For anesthesia, awakening, extubation, and anesthesia recovery time, the time from the end of surgery to the first opening of the eyes, the time to pull out the catheter, and anesthesia-recovery room stay were recorded.

For the Ramsay score, the maximum Ramsay score of each group during treatment was recorded. The scoring rules were as follows. The state of irritability was 1 point, the state of calm cooperation was 2, the lethargic state was 3, the state of drowsiness which could be awakened was 4, the state of drowsiness with nonresponse on awakening was 5, and the state of deep sleep with nonresponse on awakening was 6.

For the Riker sedation-agitation scale (SAS) score, the SAS score during surgery in each group was recorded. The scoring rules were as follows. The state that was unable to be awakened was 1 point, the state of calmness was 2, the state of relative calmness was 3, the state of calm cooperation was 4, the state of agitation was 5, the state of great agitation was 6, and the state of extreme agitation was 7.

The evaluation of nursing satisfaction is referred to the evaluation of the medical staff's service attitude, surgery, nursing process, health education, and nursing effect. Each item was 10 points, and the total score on the scale was 50 points.

For the evaluation of fMRI, the patient's brain was classified into the prefrontal lobe, temporal lobe, hippocampus, and posterior cingulate. BLOD time sequence of the areas of interest was extracted by using the resting-state fMRI Data Analysis Toolkit. After the correlation coefficient *R* of each area was calculated, the Fisher *Z* transform was performed. Then, the correlation coefficient *Z* value of each brain region was obtained.

### 2.6. Statistical Analysis

SPSS 19.0 was employed for data statistics and analysis. Mean ± standard deviation ( $\bar{x}$ ® ± *s*) was how continuous variables conforming to normal distribution were expressed, and differences among groups were compared by using the one-way analysis of variance (ANOVA). Frequency (%) was how dichotomous variables were expressed, and the differences were compared by *χ*^*2*^ test or Fisher test. Matlab2020b, SPM12, and DPABI4.3 were employed for fMRI data and analysis. The difference was statistically significant *P* < 0.05.

## 3. Results

### 3.1. Comparison of Basic Data

The basic data of patients in the control group, group A, and group B were compared (Table 1). There were insignificant differences in sex ratio, mean age, and body mass index (BMI) among the three groups(*P* > 0.05).

### 3.2. Comparison of fMRI and Functional Connectivity in Brain Regions

**Figure 1:**
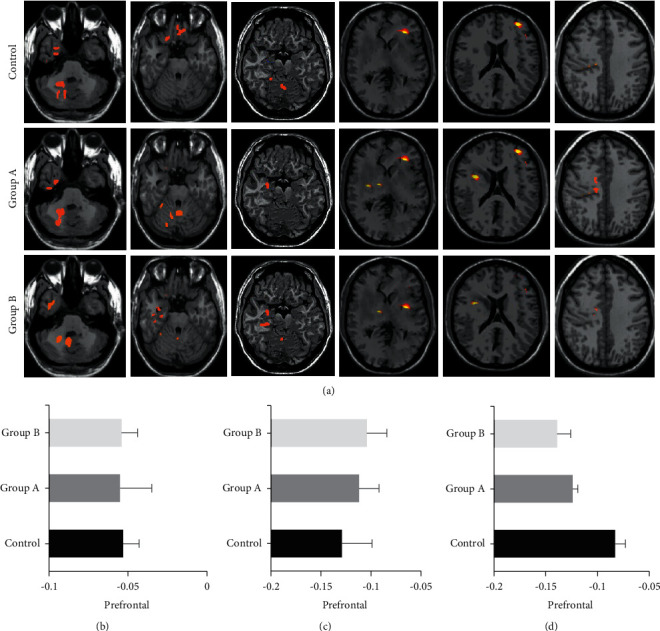
Analysis of patients' brain fMRI and functional connectivity. (a) Patients' brain fMRI images, in which the red *t*-value expressed the positive number and the blue *t*-value expressed the negative number, the horizontal images were −30 mm, −20 mm, −10 mm, +0 mm, +10 mm, and +20 mm from left to right; (b) the functional connectivity *Z* value of the prefrontal lobe; (c) the functional connectivity *Z* value of the temporal lobe; (d) the functional connectivity *Z* value of hippocampus.

Based on brain fMRI images, the local regional homogeneity (ReHo) value and the functional connectivity *Z* value of each group were compared (Figure 1). In Figure 1(a), there were significant differences in ReHo values of the posterior cerebellar lobe, lingual gyrus, occipital lobe, temporal lobe, anterior cingulate, precentral gyrus, and frontal lobe among all groups(*P* < 0.05). In Figures 1(b) and 1(c), compared with the control group, the functional connectivity *Z* values of the temporal lobe in group A and group B were increased, while those of the hippocampus were decreased, with an insignificant difference in the functional connectivity *Z* values among different brain regions in each group (*P* > 0.05).

### 3.3. Comparison of Hemodynamic Indexes

**Figure 2:**
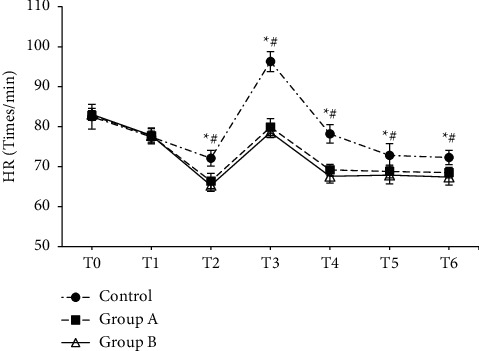
Changes in HR at different time points. *∗*: compared with group A, *P* < 0.05; #compared with group B, *P* < 0.05.

The differences in the HR indexes among the three groups at different time points were compared (Figure 2). There was no statistically considerable difference in HR among the groups at T0 and T1 time points(*P* > 0.05), but the HR was evidently increased in each group at the T3 time point. At T2, T3, T4, T5, and T6, the HR of group A and group B was notably lower than that of the control group (*P* < 0.05).

**Figure 3:**
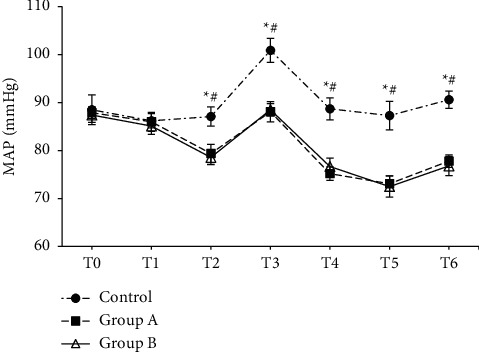
Changes of MAP at different time points. *∗*: compared with group A, *P* < 0.05; #compared with group B, *P* < 0.05.

The differences in MAP indexes among the three groups at different time points were compared (Figure 3). There was a statistically insignificant difference in MAP among the groups at T0 and T1 time points(*P* > 0.05), while MAP observably increased in each group at T3. At T2, T3, T4, T5, and T6, the MAP of group A and group B was remarkably lower than that of the control group (*P* < 0.05).

### 3.4. Comparison of Anesthesia Recovery Indexes

**Figure 4:**
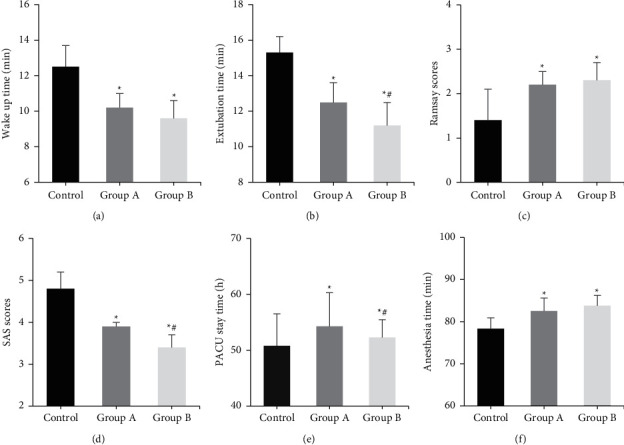
Comparison of evaluation indexes of anesthesia recovery among three groups. (a) The waking time; (b) the extubation time; (c) Ramsay's score; (d) SAS score; (e) PACU residence time; (f) maintenance time of anesthesia.  ^*∗*^: compared with the control group, *P* < 0.05; #compared with group A, *P* < 0.05.

The differences in anesthesia recovery indexes among each group were compared, including the awakening time, extubation time, the Ramsay score, the SAS score, PACU stay, and anesthesia maintenance time (Figure 4). Compared with the control group, the awakening time, extubation time, and the SAS score of patients in group A and group B were manifestly decreased, while the Ramsay score, PACU stay, and anesthesia maintenance time were all markedly increased (*P* < 0.05). Besides, compared with group A, the extubation time, SAS score, and PACU stay in group B were greatly reduced (*P* < 0.05).

### 3.5. Comparison of the Usage Amount of Anesthetic Drugs

**Figure 5:**
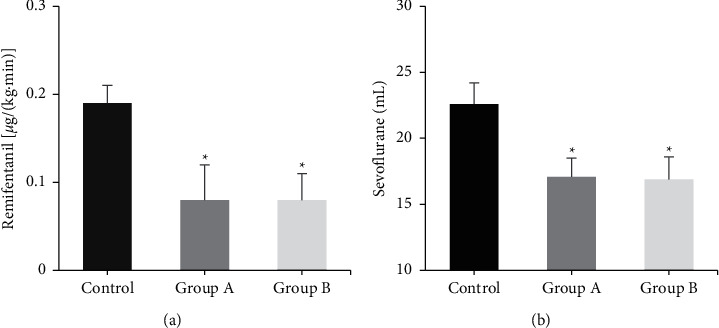
Comparison of the usage amount of anesthetic drugs among three groups. (a) dosage of remifentanil; (b) dosage of sevoflurane.  ^*∗*^Compared with the control group, *P* < 0.05.

The differences in usage amount of remifentanil and sevoflurane in each group were compared (Figure 5). The usage amount of remifentanil and sevoflurane was reduced in groups A and B compared with the control group (*P* < 0.05). There was no considerable difference in the usage amount of remifentanil and sevoflurane between group A and group B (*P* > 0.05).

### 3.6. Comparison of Hospital Stay and Nursing Satisfaction

**Figure 6:**
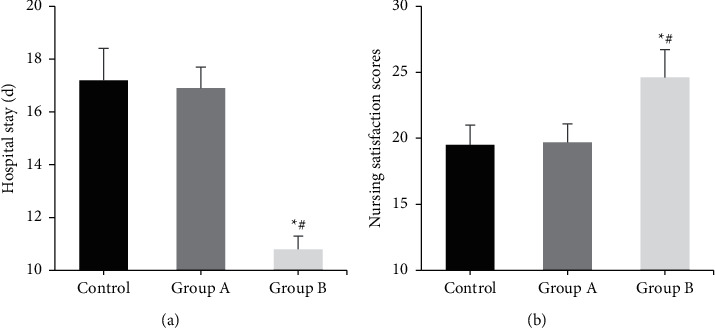
Comparison of hospital stay and nursing satisfaction among three groups. (a) Length of hospital stay; (b) nursing satisfaction score.  ^*∗*^Compared with the control group, *P* < 0.05; #compared with group A, *P* < 0.05.

The differences in length of hospital stay and nursing satisfaction scores were compared among all groups (Figure 6). Compared with the control group and group A, the length of hospital stay in group B was signally shortened, and the nursing satisfaction score was evidently increased (*P* < 0.05). The differences in length of stay and nursing satisfaction score between the control group and group A were not considerable (*P* > 0.05).

## 4. Discussion

Emergence agitation is a stress response caused by extubation during the recovery period, which occurs frequently in abdominal surgery. Emergent agitation will cause patients to be excited by the sympathetic nervous system such as accelerated heart rate, increased blood pressure, and rapid shallow breathing, which will lead to cardiovascular and cerebrovascular diseases in serious cases, thus affecting the outcome and prognosis of the disease [12, 13]. Opioids, nonsteroidal drugs, and intraoperative balloon pressure adjustment can inhibit the stress response of patients during extubation [14–16]. However, opioids can also cause such adverse reactions as respiratory depression, delayed recovery, and malignant vomiting [17]. After the general anesthesia with sevoflurane, the central nervous system of patients in the recovery stage cannot be synchronized. At that time, although the patient's consciousness has been recovered, the cortical and ascending excitatory system function of the brain area has not, which is the main reason for the agitation of patients in the recovery period after anesthesia [18]. Consequently, it is of great significance to find safe and effective drugs to prevent the emergence of agitation and maintain the stability of the circulatory system during extubation.

DEX is a class of *α*2 adrenergic receptor agonists for the nucleus ceruleus, which has the characteristics of dose-dependent sedation, analgesia, sleep aid, and inhibition of sympathetic nervous system excitation [19]. Moreover, DEX can effectively reduce the occurrence of agitation in patients after the general anesthesia surgery, with the short action time, and patients are easy to be wakened up [20, 21]. Surgical anesthesia has certain risks, and perioperative factors can cause anxiety, fear, and other psychological changes in patients. Besides, the neglect of medical staff or family members will aggravate the symptoms of emergence agitation [22]. Hence, careful nursing during the perioperative period of anesthesia plays a crucial role in reducing anxiety and fear and improving the symptoms of complications during the recovery period. The effect of DEX on brain fMRI characteristics and emergence agitation in patients who underwent general anesthesia surgery with sevoflurane under comfortable nursing intervention was explored.

Anesthesia can affect patients' cognitive function. The fMRI can intuitively evaluate the brain function of patients, with the noninvasive characteristics and high time-spatial resolution [23]. Functional connectivity is an fMRI index that is used to evaluate the synchronization of functional activity in the brain [24]. Functional connectivity of brain regions, especially the default network, is abnormal in patients with Alzheimer's disease [25]. Therefore, the prefrontal lobe, temporal lobe, and hippocampus of the default network in the resting state were compared with the posterior cingulate. The results reflected that, with different anesthesia treatments and nursing interventions, there was no significant difference in functional connectivity values of brain regions under the default network. Furthermore, there was no considerable difference in the effects of sevoflurane and DEX on the functional connectivity of the prefrontal lobe, temporal lobe, and hippocampus during a short surgery. Nevertheless, after the anesthesia with sevoflurane and DEX, the distributions of the ReHo values were manifestly different in different brain regions. During general anesthesia, patients' brain functional activities continued to change, so it was of great significance to explore the trend of changes in brain activity for the investigation of the mechanism of general anesthesia [26].

Postoperative emergence agitation is a common inappropriate behavior in patients undergoing general anesthesia surgery, which can affect the circulatory system of patients. Therefore, postoperative nursing is very important. Compared with the conventional sevoflurane anesthesia group, changes in HR and MAP were relatively stable at different time points during the perioperative period after the DEX was administrated. Continuous DEX infusion during the perioperative period was helpful in effectively inhibiting the stress response caused by intubation and maintaining the stability of perioperative hemodynamics [27]. Besides, compared with the conventional sevoflurane anesthesia group, postoperative awakening time, extubation time, the SAS score, and the usage amount of remifentanil and sevoflurane were all obviously reduced after the DEX treatment, while the Ramsay score, PACU stay, and the anesthesia maintenance time were all significantly increased(*P* < 0.05). Hence, DEX could effectively prevent the occurrence of emergence agitation in patients after general anesthesia surgery with sevoflurane [28]. Compared with DEX-assisted anesthesia patients under routine nursing, the extubation time, the SAS score, and PACU stay of patients under the comfortable nursing intervention were observably reduced, while the nursing satisfaction score was manifestly increased. Comfortable nursing is a kind of comprehensive nursing method. Through physical and mental nursing before and after the operation, the patients can have a deep understanding of the surgical process, the harm of emergence agitation, etc., so that the patients are in a relatively calm state for surgery. The observation and nursing of patients' status after general anesthesia, monitoring of patients' vital health status, timely feedback of patients' postoperative adverse reactions, and giving different degrees of psychological comfort to patients with emergence agitation can effectively reduce the threat to life and health [29]. To sum up, comfortable nursing could reduce the degree of pain of surgery, reduce the degree of agitation during postoperative recovery, and improve nursing satisfaction by adjusting the psychological state of patients during the perioperative period.

## 5. Conclusion

The effects of comfortable nursing intervention combined with DEX on brain function and the emergence agitation in patients who received the general anesthesia surgery with sevoflurane were investigated. The results showed that different anesthesia methods could change the functional connectivity of brain regions. The comfortable nursing intervention combined with DEX could effectively prevent the occurrence of emergence agitation in patients after general anesthesia surgery with sevoflurane was performed, improve patients' nursing satisfaction with medical staff, and promote the relationship between medical staff and patients. However, the functional connectivity of 3 brain regions in patients with different anesthesia methods after surgery is only evaluated in this experiment. In the future, it is necessary to evaluate the changes in functional connectivity of multiple brain regions in patients with different anesthesia methods before and after surgery. At the same time, the mechanism of DEX on emergence agitation induced by general anesthesia with sevoflurane is analyzed through an animal model experiment. In conclusion, the results of this experiment provided evidence for the clinical prevention of DEX in emergence agitation.

## Figures and Tables

**Table 1:** Comparison of basic data of patients (*n* = 66).

Group	Male (*n* (%))	Mean age (years old)	BMI (kg/m^2^)
Control group (*n* = 22)	10 (45.5)	48.3 ± 3.6	22.7 ± 2.5
Group A (*n* = 22)	11 (50.0)	47.1 ± 4.4	23.4 ± 3.1
Group B (*n* = 22)	11 (50.0)	48.7 ± 3.5	23.9 ± 2.9
*F* value	0.328	0.403	0.388
*P*	0.132	0.118	0.187

*Note.* BMI = weight (kg)/height^2^(m).

## Data Availability

The data used to support the findings of this study are available from the corresponding author upon request.
